# Design and Rationale of a Prospective International Follow-Up Study on Intensive Care Survivors of COVID-19: The Long-Term Impact in Intensive Care Survivors of Coronavirus Disease-19–AFTERCOR

**DOI:** 10.3389/fmed.2021.738086

**Published:** 2021-09-08

**Authors:** Karin Wildi, Gianluigi Li Bassi, Adrian Barnett, Mauro Panigada, Sebastiano M. Colombo, Alessandra Bandera, Antonio Muscatello, Bairbre McNicholas, John G. Laffey, Denise Battaglini, Chiara Robba, Antoni Torres, Ana Motos, Carlos M. Luna, Fernando Rainieri, Carol Hodgson, Aidan J. C. Burrell, Hergen Buscher, Heidi Dalton, Sung-Min Cho, Huimahn Alex Choi, David Thomson, Jacky Suen, John F. Fraser

**Affiliations:** ^1^Critical Care Research Group, Brisbane, QLD, Australia; ^2^Faculty of Medicine, The University of Queensland, Brisbane, QLD, Australia; ^3^Cardiovascular Research Institute Basel, Basel, Switzerland; ^4^Australian Centre for Health Services Innovation (AusHSI) and Centre for Healthcare Transformation, Queensland University of Technology QUT, Brisbane, QLD, Australia; ^5^Intensive Care, Fondazione IRCCS Ca' Granda Ospedale Maggiore Policlinico Milan, Milan, Italy; ^6^Department of Pathophysiology and Transplantation, University of Milan, Milan, Italy; ^7^Infectious Diseases Unit, Fondazione IRCCS Ca' Granda Ospedale Maggiore Policlinico Milan, Milan, Italy; ^8^Galway University Hospitals, National University of Ireland, Galway, Ireland; ^9^Anesthesia and Intensive Care, San Martino Policlinico Hospital, IRCCS for Oncology and Neuroscience, Genoa, Italy; ^10^Department of Medicine, University of Barcelona, Barcelona, Spain; ^11^Department of Surgical Sciences and Integrated Diagnostics (DISC), University of Genoa, Genoa, Italy; ^12^Servei de Pneumologia, Hospital Clinic, University of Barcelona, IDIBAPS, ICREA, CIBERESUCICOVID, Barcelona, Spain; ^13^Hospital de Clinicas, Buenos Aires, Argentina; ^14^The Alfred Hospital, Monash University, Melbourne, VIC, Australia; ^15^St. Vincent‘s Hospital Sydney, University of New South Wales, Darlinghurst, NSW, Australia; ^16^Inova Fairfax Hospital, Falls Church, VA, United States; ^17^Departments of Neurology, Neurosurgery, Anesthesia and Critical Care Medicine, John Hopkins Hospital, Baltimore, MD, United States; ^18^UTHealth, McGovern Medical School, Houston, TX, United States; ^19^Groote Schuur Hospital, University of Cape Town, Cape Town, South Africa

**Keywords:** coronavirus, SARS-CoV-2, COVID-19, intensive care unit survivors, long-term follow-up, long-term sequelae, pulmonary and cardiac impairment, health-related quality of life

## Abstract

**Background:** In a disease that has only existed for 18 months, it is difficult to be fully informed of the long-term sequelae of severe acute respiratory syndrome coronavirus-2 (SARS-CoV-2) infection. Evidence is growing that most organ systems can be affected by the virus, causing severe disabilities in survivors. The extent of the aftermath will declare itself over the next 5–10 years, but it is likely to be substantial with profound socio-economic impact on society.

**Methods:** This is an international multi-center, prospective long-term follow-up study of patients who developed severe coronavirus disease-2019 (COVID-19) and were admitted to Intensive Care Units (ICUs). The study will be conducted at international tertiary hospitals. Patients will be monitored from time of ICU discharge up to 24 months. Information will be collected on demographics, co-existing illnesses before ICU admission, severity of illness during ICU admission and post-ICU quality of life as well as organ dysfunction and recovery. Statistical analysis will consist of patient trajectories over time for the key variables of quality of life and organ function. Using latent class analysis, we will determine if there are distinct patterns of patients in terms of recovery. Multivariable regression analyses will be used to examine associations between baseline characteristics and severity variables upon admission and discharge in the ICU, and how these impact outcomes at all follow-up time points up to 2 years.

**Ethics and Dissemination:** The core study team and local principal investigators will ensure that the study adheres to all relevant national and local regulations, and that the necessary approvals are in place before a site may enroll patients.

**Clinical Trial Registration:**anzctr.org.au: ACTRN12620000799954.

## Strengths and Limitations of This Study

- This is a comprehensive international long-term (2 years) follow-up study in COVID-19 ICU survivors.
- Multi organ assessment of injury and recovery due to SARS-CoV-2 will be investigated.
- Results will potentially assist clinicians in the management of long-term COVID-19 following severe disease by defining high risk groups for follow-up and guiding personalized rehabilitation.
- Quality of recorded data will depend on physicians and research staff and in the case of high clinical workload, data completeness and accuracy could be limited.

## Introduction

A large proportion of the worldwide population has been infected by severe acute respiratory syndrome coronavirus 2 (SARS-CoV-2) during the 2020 pandemic. Whilst the vast majority of patients have survived the acute illness with minimal sequelae (1), a subgroup may suffer serious long-term sequelae (2–5), commonly referred to as “long COVID,” potentially creating a new burden to public health systems worldwide (6).

Evidence from previous investigations of the 2003 Severe Acute Respiratory Syndrome (SARS) outbreak indicates that coronaviruses substantially affect the long-term quality of life and pulmonary function in survivors. Indeed, up to one third of SARS patients had persistent pulmonary abnormalities in lung function (7, 8) and chest x-ray (9), even after 1 year from Intensive Care Unit (ICU) discharge, as well as impaired quality of life (9). Preliminary data show that at least 10% of all hospitalized COVID-19 survivors have been readmitted within 2 months (10) and presented a 33% increase in risk of developing new psychiatric or neurological diseases within the first 3 months (11, 12).

During the ongoing pandemic, up to 10 to 20% of hospitalized COVID-19 patients required admission to ICUs (1, 13), with over half of these patients requiring intubation and invasive mechanical ventilation mainly due to ARDS (1). It is well established from the literature that critical care illnesses, such as sepsis or acute respiratory distress syndrome (ARDS), are risk factors for long-term physical and neurological impairment (14–18). Similarly, COVID-19 patients in the ICU often present with severe septic shock and ARDS, ultimately requiring long-term mechanical ventilation. Thus, a large range of debilitating sequelae in those critically ill COVID-19 patients is expected (4, 19).

In response to the COVID-19 pandemic, the *COVID-19 Critical Care Consortium Observational Study* (CCCC; www.covid-critical.com) (20) was developed, an international observational cohort study, with the aim to characterize risk factors, clinical features, and outcomes of the sickest COVID-19 patients admitted to ICUs. As of May 2021, the CCCC includes 370 sites across 53 countries and has included over 10‘000 patients globally. Thus, through the COVID-19 CCC network, we aim to determine international prevalence and outcomes of patients with long-term COVID-19 to elucidate the pathophysiology of organ injury and recovery.

## Materials and Methods

### Study Design and Setting

The Long-term imp*a*ct in in*te*nsive ca*r*e survivors of *cor*onavirus disease-19 (AFTERCOR) study is an international multi-center, prospective interventional study of patients who have been infected by SARS-CoV-2, developed symptomatic COVID-19 and were admitted to an ICU because of this disease and its complications. Patients will be studied from time of hospital discharge up to 24 months. Information will be collected on demographics, co-existing illnesses before ICU admission, severity of illness during ICU admission and post-ICU quality of life and organ dysfunction/recovery.

### Eligibility

Patients qualified for enrolment if the following conditions were fulfilled: 1) laboratory-confirmed SARS-CoV-2 infection by real-time polymerase chain reaction and/or next generation sequencing 2) written informed consent from the patient at the time of discharge from the hospital or first follow-up 3) age ≥18 years 4) ICU admission diagnosis of COVID-19 infection and/or any of its related complications, i.e., respiratory failure, sepsis and septic shock, acute liver, kidney or cardiac injury, secondary infection, rhabdomyolysis, coagulation disorders or disseminated intravascular coagulation. Previous enrolment into the CCCC observational study is favorable but not mandatory. Exclusion criteria are 1) pregnancy at time of infection 2) inability to complete long-term follow-up, due to logistical problems 3) paralysis due to pre-existing neurological condition before being admitted to hospital for COVID-19 4) history of pulmonary resection 5) previous pulmonary transplant 6) documented advanced neurologic disorder which precludes 6-min walking test 7) documented psychiatric disease for which the patient is unable to carry out the interviews.

### Screening and Recruitment

Suitable patients within the AFTERCOR network will be identified by study personnel and approached for written informed consent while in recovery after ICU discharge or at the first scheduled follow-up assessment. Informed consent will be obtained directly from the patient.

### Enrolment and Participating Sites

Study enrolment began on January 1st 2021. Currently, 14 centers in 11 countries (Italy, Spain, Ireland, Austria, United States, Australia, South Africa, Japan, Argentina, Brazil, Colombia) are participating whilst more sites are being recruited. Co-enrolment in other studies is possible as long as these are observational only.

### Outcome Measures

Follow-up assessments will be scheduled as outpatient clinic visits at 3, 6, 12, 18, and 24 months post-hospital discharge (Table 1).

**Table 1:** Planned assessments per follow-up time point.

	**3 months**	**6 months**	**12 months**	**18 months**	**24 months**
Frailty score	x				
**Quality of life, psychiatric symptoms**					
SF-36	x	x	x	x	x
PHQ-9	x	x	x	x	x
**Pulmonary assessment**					
SGRQ	x	x	x	x	x
Spirometry^†^	x	x	x	x	x
aBGA^†^	x	x	x	x	x
Body phletysmography^†^	x	x	x	x	x
Airway resistance^†^	x	x	x	x	x
6-min WT^†^	x	x	x	x	x
Chest x-ray^†^	x	x	x		x
CT^*^		x			
MoCA	x	x	x	x	x
Blood analysis	x	x	x	x	x
Echocardiography^*^	x		x		x

*SF-36, Short Form Health Survey 36; SGRQ, St George Respiratory Questionnaire; PHQ-9, Patient Health Questionnaire-9; aBGA, arterial blood gas analysis; WT, walking test; MoCA, Montreal cognitive Assessment*.

† *Assessments will be repeated upon normalization*.

* *Only if CT and/or echocardiography was undertaken while on ICU*.

#### Pulmonary Assessment

Any admission for respiratory failure or a new respiratory infection since ICU discharge or last follow-up visit will be recorded. The St. George's respiratory questionnaire will be used to measure the subjective self-reported quality of life associated with chronic respiratory diseases (7, 21). Basic spirometry pre- and post-bronchodilation according to the standards of the American Thoracic Society (22) will be performed at every follow-up visit or until normalization. If in the subsequent arterial blood gas analysis, the partial pressures of oxygen and carbon dioxide are <75 mmHg or >42 mmHg, respectively by breathing room air, we will extend assessments by body plethysmography, measurement of airway resistance and gas exchange.

A standardized 6-minute walking test will be carried out following recommendations from the European Respiratory Society (23). A conventional chest x-ray will be performed and additionally, in patients who underwent a chest computer tomography scan during their ICU stay, a follow-up computer tomography scan will be done at 6 months to assess persisting changes.

Pulmonary function tests, arterial blood gas analysis, chest x-ray and the 6-minute walking test will be performed at every follow-up visit or until normalization of the test results. If a result is deemed to be in the normal range for the patient, the test will not be repeated during the following visits.

#### Assessment of Health-Related Quality of Life

The Short Form Health Survey, a patient-reported questionnaire consisting of 36 items, will be used to assess health-related quality of life (24, 25). Symptoms suggestive of depression will be screened through the Patient Health Questionnaire-9, a self-administered diagnostic instrument which has been validated for use as a screening tool in primary care (26). Additionally, we will assess whether the patient was able to return to work and under what conditions, which is an important economic and well-being outcome.

At the 3 month post-ICU discharge follow-up visit, we will determine the patient's clinical frailty score before ICU admission, through recollection of his/her clinical status by the patient and/or relatives. The used score ranges from 1 (very fit) to 9 (terminally ill) (27).

#### Assessment of the Dynamics of Kidney, Cardiac, Liver, and Neurological Injury and Recovery

Any hospital admission for neurological decompensation, cardiac, renal or liver failure since ICU discharge or last follow-up visit will be recorded, as well as the need for hemodialysis in case of renal failure. Liver, heart and renal biomarkers (e.g., creatinine, estimated glomerular filtration rate, liver function tests, brain-natriuretic peptide) and full blood count will be determined in a blood sample at every follow-up visit. Potential cognitive impairment will be screened through the Montreal cognitive assessment (28). In patients who underwent an echocardiography during their ICU stay, a follow-up focussed echocardiography will be performed at 3, 12, and 24 months or until normalization.

#### Vaccination Status

As new evidence indicates that the COVID-19 vaccination can possibly influence the outcome of Long-COVID positively, details regarding received vaccinations during the follow-up period will be recorded (all currently available vaccines, date of administration, number of doses). Additionally, if a concomitant influenza vaccination was given, this will be recorded too.

### Parameters Collected Regarding COVID-19 Related ICU Stay

If the patient has been enrolled in CCCC previously, the data collected during their ICU stay regarding demographics, ethnicity, presence of predefined comorbidities, severity of critical illness, clinical course during ICU stay and antiviral/antibiotic medications as outlined below will be available for linkage to the follow-up assessments. If the patient has not been enrolled in CCCC, the above information will be collected by the enrolling study team. We will record information about the following possible variants of concern (VOC) or variants of interest (VOI) identified in the patient upon ICU admission: Alpha-B.1.1.7, identified in UK Sept 2020; Beta-B.1.351, identified in South Africa May 2020; Gamma-P.1, identified in Brazil Nov 2020; Delta-B.1.617.2, identified in India Oct 2020; Epsilon-B.1.427/B.1.429, identified in USA Mar 202; Zeta-P.2, identified in Brazil Apr 2020; Eta-B.1.525, identified in Multiple Countries Dec 2020; Theta-P.3, identified in Philippines Jan 2021; Iota-B.1.526, identified in USA Nov 2020; Kappa-B.1.617.1, identified in India Oct 2020; Lambda-C.37, identified in Peru Dec 2020. Detailed information about medical comorbidities, including pre-existing immunosuppression, malnutrition and dementia will be collected. Relevant medication pre-ICU admission will be also recorded, especially angiotensin-converting enzyme inhibitors and/or angiotensin II receptor blockers, non-steroidal anti-inflammatory drugs, corticosteroids or other immunosuppressant drugs, antibiotics and/or antivirals. The last full blood count, biochemistry panel and arterial blood gas analysis while on ICU will be transferred into the electronic database. In terms of supportive treatment, we will record oxygen therapy, non-invasive and invasive ventilation, prone positioning, administration of inhaled nitric oxide, tracheostomy, extracorporeal membrane oxygenation support, cardiac-assist devices and renal replacement therapy as well as the duration in days for every intervention. Collected data regarding pharmacological treatment includes inotropic and vasoactive agents, antiviral or COVID-19 targeted treatment [f.e. ribavirin, lopinavir/ritonavir, remdesivir, interferon alpha and/or beta, (OH-) chloroquine], antibiotic and antifungal agents, corticosteroids and anticoagulation (all in detail regarding preparation, dose, route of administration and duration). In addition, any of the following complications during the ICU stay will be recorded: viral pneumonia/pneumonitis, bacterial pneumonia, acute respiratory syndrome, pneumothorax, pleural effusion, cryptogenic organizing pneumonia, bronchiolitis, cardiac arrest, myocardial infarction, myocarditis/pericarditis, endocarditis, cardiomyopathy, congestive heart failure, seizure, stroke/cerebrovascular event, meningitis/encephalitis, bacteremia, coagulation disorder, DIC, pulmonary embolism, deep vein thrombosis, anemia, rhabdomyolysis, acute renal failure, gastrointestinal hemorrhage, pancreatitis, liver dysfunction, hypo- and hyperglycemia. If a chest x-ray, echocardiography and/or CT thorax was performed while the patient was on ICU, the date of the exam and the detailed results will be recorded. The detailed AFTERCOR case report form can be found at the official AFTERCOR website (https://www.aftercorstudy.com).

### Data Management and Confidentiality

The case report form (CRF) will be available as a paper and electronic CRF (eCRF) for data collection. All documents will be stored securely to maintain confidential conditions. The site Principal Investigator is responsible for securely locking away hard copy documentation and ensuring electronic information is password protected. Access to secure files will only be granted to selected study personnel, this includes access to the online database (REDCap, hosted by the University of Queensland). All data will be cleaned at regular intervals to ensure accuracy and maintain high data quality. On all trial-specific documents, other than the signed consent form, the participant will be referred to by a unique trial-specific patient identifier code, including the electronic database. The electronic database has been developed as a web-based tool and can be accessed online, all entered data is completely de-identified.

### Sample Size Calculation

This is an observational study that will examine multiple research questions. We aim for enrolment of at least 1,000 patients: this sample size will provide good power to detect differences between groups, for example it gives a 90% power to detect a standardized difference of 0.15 between two equally sized groups (using a two-sided alpha of 0.05).

### Statistical Considerations

Summary statistics will be used to describe the sample characteristics at ICU discharge and plots to summarize outcomes over time for both individual patients and averages by groups. Patient trajectories will be plotted over time following discharge to examine patterns in recovery for the key variables of quality of life and lung health. Mean trajectories will be used to summarize the typical trajectory. Latent class analysis will be used to determine whether there are distinct groups of patients in terms of recovery, e.g., fast and slow recovery groups, and whether these groups have particular clinical characteristics. Multivariable regression analyses will be used to examine associations between baseline characteristics and severity variables upon admission and discharge in the ICU, and how these impact outcomes at 3, 6, 12, 18, and 24 months after ICU discharge. The residuals of the models will be checked for multi-modality, skew and outliers. Models will also be checked for collinearity and influential values. Participant drop-out rates will be displayed over time and we will examine whether patient characteristics at baseline predict drop-out. Item-missing data will be summarized, and we may use multiple imputation methods as a sensitivity analysis to compensate for missing data. Long-term survival will be plotted using a Kaplan–Meier plot with censoring for patients lost to follow-up. A multivariable survival model will be used to examine which variables predict survival. We will use the lasso to select the important subset of predictors from a larger set. All analysis will be performed with R (R Foundation for Statistical Computing, Vienna, Austria).

The results of this study will be reported as according to the STROBE guidelines (29).

### Ethical Principles and Guidelines

The Chief Investigators and study management team will be responsible for ensuring that the study is conducted in accordance with the protocol, ethical principles of the Declaration of Helsinki (June 1964, most recently amended in October 2013) and the most recent, relevant ethical conduct of research guidelines published in the country of the participating site.

Each participating site will require ethics approval for the protocol, data collection and any other study documents relevant to their region. Site Chief Investigators will ensure that (a) ethics approval has been granted prior to commencing the study and (b) all local regulatory requirements are addressed. The study management team, based in Brisbane, Queensland, Australia will work closely with the local Chief Investigators to ensure that the conduction of the study adheres to all relevant national regulations and the Principles of Good Clinical Practice.

### Dissemination

The results of this study will be presented at international conferences and prepared for publication in peer-reviewed journals upon completion of the study.

## Discussion

This study aims to provide a comprehensive understanding of the long-term impact of COVID-19 in patients who have been admitted to ICU. The primary outcome of the study is to extensively characterize physical, mental and psychosocial consequences post ICU discharge. Secondary outcomes include long-term health and social-economic cost of COVID-19, risks for medical sequelae and long-term survival.

The emerging data and evidence suggest that a significant proportion of critically ill COVID-19 patients will present persistent debilitating symptoms, organ dysfunctions and compromised quality of life post-hospital discharge (4, 6, 12). Results obtained from this study will assist clinicians and healthcare providers to better identify high risk patients who are recovering from COVID-19, then to target interventions that improve their post-COVID-19 quality of life, facilitating a quicker return to productive life. Better understanding of the long-term impact of COVID-19 through data collected in this study will help and inform governments and policy-makers to better allocate resources.

## Limitations

As this is a long study with multiple and extensive follow-up assessments, a high-drop-out rate of patients is expected. The statistical assumptions will be corrected for this potential confounder of results.

## Data Availability Statement

The original contributions presented in the study are included in the article/supplementary material, further inquiries can be directed to the corresponding author/s.

## Ethics Statement

This study involving human participants, was reviewed and approved by the Human ethics committee at The Prince Charles Hospital in Brisbane (Australia), Granda Ospedale Maggiore Policlinico in Milan (Italy), Galway University Hospitals (Ireland), John Hopkins Hospital in Baltimore (USA), UTHealth in Houston (USA), Hospital Clinic in Barcelona (Spain) and Hospital de Clinicas in Buenos Aires (Argentina). All enrolled patients/participants will provide their written informed consent to participate in this study.

## Author Contributions

KW, GL, ABar, MP, SC, ABan, AM, BM, JL, DB, CR, AT, AM, CL, FR, CH, AJCB, HB, HD, S-MC, HC, DT, JS, and JF planned the study. KW and GL are the chief investigators of the study and MP, BM, JL, DB, CR, AT, CL, CH, HB, S-MC, HC, and DT are the local chief investigators. All authors revised and edited the final version of this manuscript and approved the submission.

## Funding

The AFTERCOR study is supported by research grants from the Wesley Medical Research Foundation (Brisbane, Australia) for the Australian sites and Gilead (USA) for two US American sites.

## Conflict of Interest

KW has received research funding from the FAG Basel (Freiwillige Akademische Gesellschaft), the Julia und Gottfried Bangerter-Rhyner-Stiftung, the Prince Charles Hospital Foundation, the University of Queensland and The Wesley Medical Research Foundation, all outside of the submitted work. GL has received research funds, through his affiliated institution from Fisher & Paykel. JL has received consulting fees from Baxter Healthcare and from GlaxoSmithKline. AT received a grant from the Instituto de Salud Carlos III (ISCII) (CiberesUCICOVID study). JS received an Advance Queensland Industry Research Fellowship. JF received a fellowship from the Queensland Department of Health. The remaining authors declare that the research was conducted in the absence of any commercial or financial relationships that could be construed as a potential conflict of interest.

## Publisher's Note

All claims expressed in this article are solely those of the authors and do not necessarily represent those of their affiliated organizations, or those of the publisher, the editors and the reviewers. Any product that may be evaluated in this article, or claim that may be made by its manufacturer, is not guaranteed or endorsed by the publisher.

## Abbreviations

CCCC: COVID-19 Critical Care Consortium
COVID-19: Coronavirus Disease 2019
CRF: Case Report Form
ICU: Intensive Care Unit
REDCap: Research Electronic Data Capture
SARS: Severe Acute Respiratory Syndrome
SARS-CoV-2: Severe Acute Respiratory Syndrome Coronavirus-2
VOC: variants of concern
VOI: variants of interest.
